# Zinc finger protein 91 loss induces cardiac hypertrophy through adenosine A1 receptor down‐regulation under pressure overload status

**DOI:** 10.1111/jcmm.15630

**Published:** 2020-07-17

**Authors:** Xiangqi Wu, Wei You, Zhiming Wu, Fei Ye, Shaoliang Chen

**Affiliations:** ^1^ Division of Cardiology Nanjing First Hospital Nanjing Medical University Nanjing China

**Keywords:** adenosine A1 receptor, cardiac hypertrophy, N6‐Cyclopentyladenosine, zinc finger protein 91

## Abstract

The function of *zfp91* is mainly studied in vitro, but there is no study in vivo. Accumulative data suggest that *zfp91* may be an important gene to regulate all aspects of human response. However, there are no data to date about the function of *zfp91* on cardiac homeostasis. Thus, we aimed to observe the role of *zfp91* gene in mouse cardiomyocytes on myocardial homeostasis and related mechanisms under pressure overload. In the study, *zfp91* mRNA and protein levels were significantly reduced in TAC‐operated WT mice as compared with controls. Genetic ablation of *zfp91* dramatically led to pathological cardiac dysfunction and hypertrophy after transverse aortic constriction (TAC). Adenosine A1 receptor (Adora1) mRNA and protein expressions were significantly down‐regulated in the heart of *zfp91*‐deletion mice with TAC. Zfp91 overexpression reversed isoproterenol‐induced cardiomyocyte hypertrophy, which was abolished by selective Adora1 antagonist. Dual‐luciferase reporter and ChIP‐qPCR assays indicated that zfp91 acted on Adora1 promoter through its binding site. Last, Adora1 agonist rescued heart dysfunction and cardiac hypertrophy in z*fp91* loss mice after TAC. Zfp91 may transcriptionally regulate Adora1 expression in the heart, which mainly maintained cardiac homeostasis under pressure overload status. It will provide a new approach to treat cardiac hypertrophy.

## 1 INTRODUCTION

With development of world's economy and improvement of people's living standards, incidence of various cardiovascular diseases is increasing year by year, which seriously affects quality of people's health and leads to high mortality.^1^ Cardiac hypertrophy is a kind of cardiovascular disease complication with high incidence, which is mainly manifested in enlargement of cardiomyocytes, coarsening and lengthening of cardiac fibres, thickening of ventricular wall and enlargement of cardiac volume. With development of disease course, cardiac hypertrophy will lead to the reduction of cardiac cavity, cardiomyocyte disappearance and myocardial fibrosis, which will lead to heart dysfunction, the obstruction and disorder of electrocardiographic conduction, and eventually lead to heart failure and sudden death.^2^, ^3^, ^4^, ^5^

Although there are extensive and in‐depth studies on the mechanism of myocardial hypertrophy, the concrete mechanism is still unclear. It has been shown that the activation of some transcription factors, including GATA binding protein 4 (GATA4) and myocyte enhancer factor 2 (MEF2), leads to heart remodelling and hypertrophy.^6^, ^7^ Zinc finger transcription factors can be used as transcriptional activators or inhibitors to regulate the expression of downstream target genes, so as to produce a wide range of molecular effects in maintaining cell homeostasis, regulating the expression of a variety of anti‐inflammatory and/or pro‐inflammatory factors, as well as cell proliferation, migration and apoptosis and other physiological or pathological processes.^8^, ^9^ The transcription factor zinc finger protein 91 (zfp91) belongs to zinc finger protein family. It contains the classical domain C2H2 which binds to nucleic acid, and regulates the expression of many downstream target genes. Until now, the function of zfp91 is mainly studied in vitro, but there is no study in vivo. Accumulative data suggest that zfp91 may be an important gene to regulate all aspects of human response.^10^ Once it mutates, it may induce a variety of diseases.

However, there are no data to date about the function of zfp91 on regulating cardiac homeostasis. Therefore, in the present study we knock out *zfp91* gene in mouse cardiomyocytes to observe its role on myocardial homeostasis and related mechanisms under pressure overload status.

## 2 MATERIALS AND METHODS

### 2.1 Mice, transverse aortic constriction (TAC) model and drug therapy

Mice with a C57BL/6 genetic background were housed in groups with 12‐hour dark/light cycles and with free access to food in accordance with the regulations on mouse welfare and ethics of Nanjing Hospital affiliated to Nanjing Medical University (Nanjing, China). The animal protocol was reviewed and approved by the Ethics Committee of Nanjing Hospital Affiliated to Nanjing Medical University. Cervical dislocation was a method to provide the mouse with a fast and painless death. Zinc finger protein 91 (*zfp91*)‐floxed mice were maintained on a C57BL/6 genetic background. To delete *zfp91* in cardiomyocytes, *zfp91*‐floxed mice were crossed with a‐myosin heavy chain (*αMHC*)‐Cre mice, and the progenies were genotyped by PCR. DNA primers for *zfp91* genotyping were as follows: 5′‐AGTTAGGCTTTGCTTTGA‐3′ and 5′‐ATAATCAGGCTTAGGTGC‐3′ to amplify wild‐type and 5′ floxed alleles, respectively. PCR products for wild‐type and floxed alleles were 223 bp and 260 bp, respectively.

TAC was generated following a method previously reported in mice with slight modifications.^11^ Briefly, 2‐month‐old mice were anaesthetized intraperitoneally with pentobarbital sodium (30‐50 mg/kg). Before TAC, we measured mice the aortic arch diameter by echocardiography. Furthermore, TAC procedure was only done by an experienced operator. An endotracheal tube was introduced into the trachea, and a volume‐cycled rodent respirator (model 683; Harvard Apparatus) provided positive pressure ventilation at 2‐3 mL per cycle with a respiratory rate of 120 cycles per minute. After the left parasternal skin incision, the transverse aorta was exposed between the thymus gland, and a 7‐0 silk suture was placed around a 27‐gauge blunt‐ended needle on the transverse aorta, which was removed immediately to yield a narrowing 0.4 mm in diameter. The sham‐operation mice were performed using the same procedure without the transverse aortic ligation. The chest wound was closed with a 7‐0 silk suture.

First, to investigate the phenotype of *zfp91* loss in cardiomyocyte under pathological stress in vivo, 2‐old‐month mice were randomly divided into control *zfp91^F/F^* mice (n = 8), control *zfp91^F/F^*; *αMHC*‐Cre mice (n = 8), *zfp91^F/F^* mice with TAC (n = 8) and *zfp91^F/F^*; *αMHC*‐Cre mice with TAC (n = 8). These mice were under the status of pressure overload for 6 weeks to test the phenotype and collect the samples. Then, to validate the target of zfp91, mice were randomly divided into control *zfp91^F/F^* mice (n = 7), control *zfp91^F/F^*; *αMHC*‐Cre mice (n = 7), *zfp91^F/F^* mice with TAC (n = 7), *zfp91^F/F^*; *αMHC*‐Cre mice with TAC (n = 7), *zfp91^F/F^* mice after TAC treated with N6‐Cyclopentyladenosine (CPA) (n = 7) and *zfp91^F/F^*; *αMHC*‐Cre mice after TAC treated with CPA (n = 7). CPA (Abcam) was administrated to mice (*zfp91^F/F^* and *zfp91^F/F^*; *αMHC*‐Cre) i.p. at a dose of 2 mg/kg of bodyweight for 6 weeks after TAC surgery.

### 2.2 Cardiac function assessment by echocardiography

On day 42 following the TAC induction, an echocardiographic examination was performed using a Vevo 770 UBM system or a Vevo 2100 UBM system (Visual Sonics), equipped with a 30‐MHz transducer, which was used for non‐invasive transthoracic echocardiography. Two‐dimensional guided M‐mode tracings were recorded. The internal diameter of the LV in the short‐axis plane was measured at end diastole and end systole from M‐mode recordings just below the tips of the mitral valve leaflets. After measurement, left ventricular end‐diastolic volume (LV Vol;d) as indicative of heart diastolic function were determined. Three indexes of heart systolic function including left ventricular end‐systolic volume (LV Vol;s), LV fractional shortening (LVFS) and LV ejection fraction (LVEF) were calculated. LV mass and LV mass corrected were two indexes of heart weight.^12^

### 2.3 Western blot analysis and quantitative real‐time PCR

Western blot analyses were performed as previously reported.^13^ Left ventricles of mice were dissected and snap‐frozen in liquid nitrogen. Tissue lysates or cell samples were prepared in lysis buffer (20 mmol/L Tris, 150 mmol/L NaCl, 10% glycerol, 20 mmol/L glycerophosphate, 1% NP40, 5 mmol/L ethylenediaminetetraacetic acid (EDTA), 0.5 mmol/L ethylenebis (oxyethylenenitrilo) tetraacetic acid (EGTA), 1 mmol/L Na3VO4, 0.5 mmol/L phenylmethanesulphonyl fluoride (PMSF), 1 mmol/L benzamidine, 1 mmol/L DL‐Dithiothreitol (DTT), 50 mmol/L NaF, 4 μmol/L leupeptin, pH = 8.0). Equal amounts of total proteins (50 μg) were resolved by 10% sodium dodecyl sulphate‐polyacrylamide gel electrophoresis (SDS‐PAGE) and transferred to polyvinylidene fluoride (PVDF) membranes (Millipore). Membranes were blocked with 5% non‐fat milk in Tris‐buffered saline Tween (TBST) (50 mmol/L Tris, 150 mmol/L NaCl, 0.5 mmol/L Tween‐20, pH = 7.5) and then incubated overnight with primary antibodies. Zfp91, adenosine A1 receptor (A1R) and Flag were produced from Abcam, Abcam and Proteintech respectively. GAPDH and HRP‐linked secondary antibodies were purchased from Bioworld Technology and Thermo Scientific, respectively. ImageJ software (NIH) was used to perform densitometric analysis.

Total RNA was extracted from mouse heart tissue with TRIzol reagent (Invitrogen) according to the manufacturer's instruction. The concentration of RNA in each sample was measured with NanoDrop spectrophotometer (Thermo). Total RNA (2 μg) was reversely transcribed to cDNA by using Reverse Transcription Kit (Takara). Real‐time PCR was performed using SYBR Green PCR Master Mix (Takara). GAPDH gene was used as the internal control. The primers used are as follows: *zfp91*: forward: 5′‐CCTCCCTCAGGAAGTTTCCATT‐3′, reverse: 5′‐TAATGCCACCGGGAGACTGATG‐3′; *Adora1*: forward: 5′‐TGTGCCCGGAAATGTACTGG‐3′, reverse: 5′‐TCTGTGGCCCAATGTTGATAAG‐3′; and *GAPDH*: forward: 5′‐AGGTCGGTGTGAACGGATTTG‐3′, reverse: 5′‐GGGGTCGTTGATGGCAACA‐3′.

### 2.4 Histology and immunofluorescence staining

The protocols for Masson's staining, haematoxylin‐eosin staining (HE) and immunofluorescence (IF) were performed as reported previously.^13^ Briefly, heart samples were first washed with ice‐cold PBS and then fixed in 4% paraformaldehyde at 4°C. The samples were processed successively by (a) a 30‐min washing in PBS at 4°C; (b) 15 minutes each in 30%, 50%, 75% and 85% ethanol, and then 2 × 10 minutes of incubation in 95% and 100% ethanol at room temperature (RT); (c) 3 × 10 minutes of incubation in xylene at RT; (d) 20 minutes of incubation in paraffin/xylene (1:1) at 65°C; and (e) 3 × 30 minutes of incubation in fresh paraffin at 65°C. The processed heart samples were embedded in paraffin and sliced into a thickness of 6 μm, and then the sections were stained for Masson and HE.

Immunofluorescence (IF) staining was performed by using anti‐wheat germ agglutinin (WGA) (Abcam) antibody at 4°C room overnight. Goat anti‐rabbit IgG (Abcam) diluted in PBS was then incubated for 2 hours at room temperature. Fluorescence microscopy images were obtained with a Research Fluorescence Microscope (Olympus America Inc) equipped with a digital camera. Images were collected and recorded by using Adobe PhotoshopR 5.0 (Adobe Systems Inc) on an IBM R52 computer (IBM). Fibrotic area and cardiomyocyte area were measured and averaged after calculating in 5 high‐power fields.

### 2.5 Microarray analysis

Three hearts (left ventriculars) were dissected from each group of control and zfp91‐deletion mice with TAC for 1 week, and total RNA was isolated from these hearts. In total, six mRNA samples were applied for microarray analysis (Affymetrix Mouse 430 2.0 Array, Shanghai Biotechnology). The data were analysed with the eBioService System (Shanghai Biotechnology).

### 2.6 Construction of plasmids and recombinant virus vectors

To construct expression plasmid, *zfp91* cDNA was amplified by RT‐PCR and its C‐terminal fusion Flag was constructed on pcDNA3.1 eukaryotic expression vector, which was cloned into the pcDNA3.1‐*zfp91*‐FLAG plasmid. Additionally, 200 bp near the predicted binding site of *Adora1* gene was constructed into pGL3 basic vector. At the same time, the mutant plasmid of *Adora1* gene was constructed: *Adora1* promoter—wild type (sense strand): TAGGGGAGGAGACGGAGGGCGACGAGGGAGGGGCCCGCGGGTGCCGCCGCCGCCCCCGCCCCCGCCCCCTCCCCCTCCCGGTGTGCGGAGCCCGATTGTC and *Adora1* promoter—mutant (sense strand): TAGGGGAGGAGACGGAGGGCGACGAGGGAGGGGCCCGCGGGTGCCGCCGCCGCCCCCGCCCCCGAACGTAGGTACTGCCGGTGTGCGGAGCCCGATTGTC. Also, recombinant adenovirus that expressed full‐length mouse *zfp91* cDNA with Flag tag (Ad‐ZFP91) was constructed. Viruses were amplified and titrated in 293 cells according to the manufacturer's instruction. Adenovirus containing empty plasmid (Ad‐con) served as control.^14^

### 2.7 Cardiomyocyte culture and adenovirus transfection

Ventricular myocytes were prepared from Sprague‐Dawley rats (2‐3 days after birth) and cultured on Permanox chamber slides (Thermo Scientific Nunc) coated with 1% gelatin and 0.0015% laminin solution, as previously described.^15^ First, these cells were transfected with Ad‐con or Ad‐ZFP91. Then, cardiomyocytes transfected with Ad‐con were intervened with dimethyl sulphoxide and isoproterenol (ISO) (20 μmol/L) plus dimethyl sulphoxide, respectively. Meanwhile, cardiomyocytes transfected with Ad‐ZFP91 were intervened with isoproterenol (ISO) (20 μmol/L) plus dimethyl sulphoxide or isoproterenol (ISO) (20 μmol/L) plus selective Adora1 antagonist 8‐Cyclopentyl‐1,3‐dimethylxanthine (CPT) (Abcam) (1 μmol/L). This process lasted 2 days. Finally, these cells were collected for cardiac troponin T (cTnT) staining and Western blot analysis.

### 2.8 Dual‐luciferase reporter assay and chromatin immunoprecipitation (ChIP)‐qPCR analysis

293 cell lines were inoculated in 24‐well culture plate the day before transfection, and the cell density was 70%‐80% at the time of transfection. 2 μL lipo2000 was diluted to 50 μL serum‐free DMEM, plasmids were diluted to 50 μl serum‐free DMEM and incubated at room temperature for 20 minutes, and serum‐free DMEM was supplemented to 100 μL. The culture medium from the dish was removed; 200 μL of the transfection complex prepared in the previous step was added and cultured at 37°C for 5 hours. The medium was removed and changed to 0.5 mL complete medium, and cultured at 37°C for 48 hours and transfected repeatedly for 5 times. Transfection plasmid combination was as follows: ① pCDNA3.1 + pGL3‐Basic + pRL‐TK; ② zfp91 + pGL3‐Basic + pRL‐TK; ③ pCDNA3.1 + Adora1‐WT + pRL‐TK; ④ zfp91 + Adora1‐WT + pRL‐TK; ⑤ pCDNA3.1 + Adora1‐MT + pRL‐TK; and ⑥ zfp91 + Adora1‐MT + pRL‐TK. Lysate cells were collected 10 000 *g* of lysate and centrifuged for 5 minutes, and supernatant was taken as test solution and then operated according to the instructions of double luciferase test kit. In the case of taking the marine luciferase as internal reference, the RLU value determined by the firefly luciferase was divided by the RLU value determined by the marine luciferase. According to the ratio obtained, the activation degree of target reporter gene was compared among different samples.^14^

ChIP was carried out according to the instructions of the EZ‐ChIP™ Chromatin Immunoprecipitation Kit (Millipore). After ChIP, the DNA precipitated by the anti‐Flag antibody was detected with qPCR, which was conducted in a final volume of 25 μL containing 12.5 μL of 2 × SYBR Mix, Taq DNA Polymerase (Takara), 1 μL each of forward primer and reverse primers (10 μmol/L), and 6 μL of DNA template under the following conditions: The template was first denatured at 94°C for 10 minutes and then subjected to 50 cycles of amplification (94°C for 20 seconds, 60°C for 1 minutes), 95°C for 2 minutes, 72°C for 1 minute, 95°C for 30 seconds, and 55°C for 10 seconds (repeat 80 times), 30°C for 1 minutes. After PCR, relative data quantification was performed using the 2^−ΔΔCt^ method, and the result was calculated in the form of %Input, which was given by the following formula: %Input = 2^(Ctinput−CtChIP)^ × input dilution factor × 100. A segment of the *Adora1* promoter containing the *Zfp91*‐binding sites was amplified using the primers 5′‐CTCACACTGAATCACTTCCTTTGTTTAG‐3′(forward) and 5′‐GGTAGCCAGCCGAGACTCC‐3′(reverse).^16^

### 2.9 Statistical analysis

Data are presented as means ± SEM values. Statistical analyses were performed using SPSS version 20 (SPSS Inc). Tukey's post hoc test was used between two groups following a *P* < .05 of one‐way ANOVA. A *P* < .05 was considered statistically significant.

## 3 RESULTS

### 3.1 Generation of cardiomyocyte‐specific *Zfp91*‐deletion mice

**FIGURE 1:**
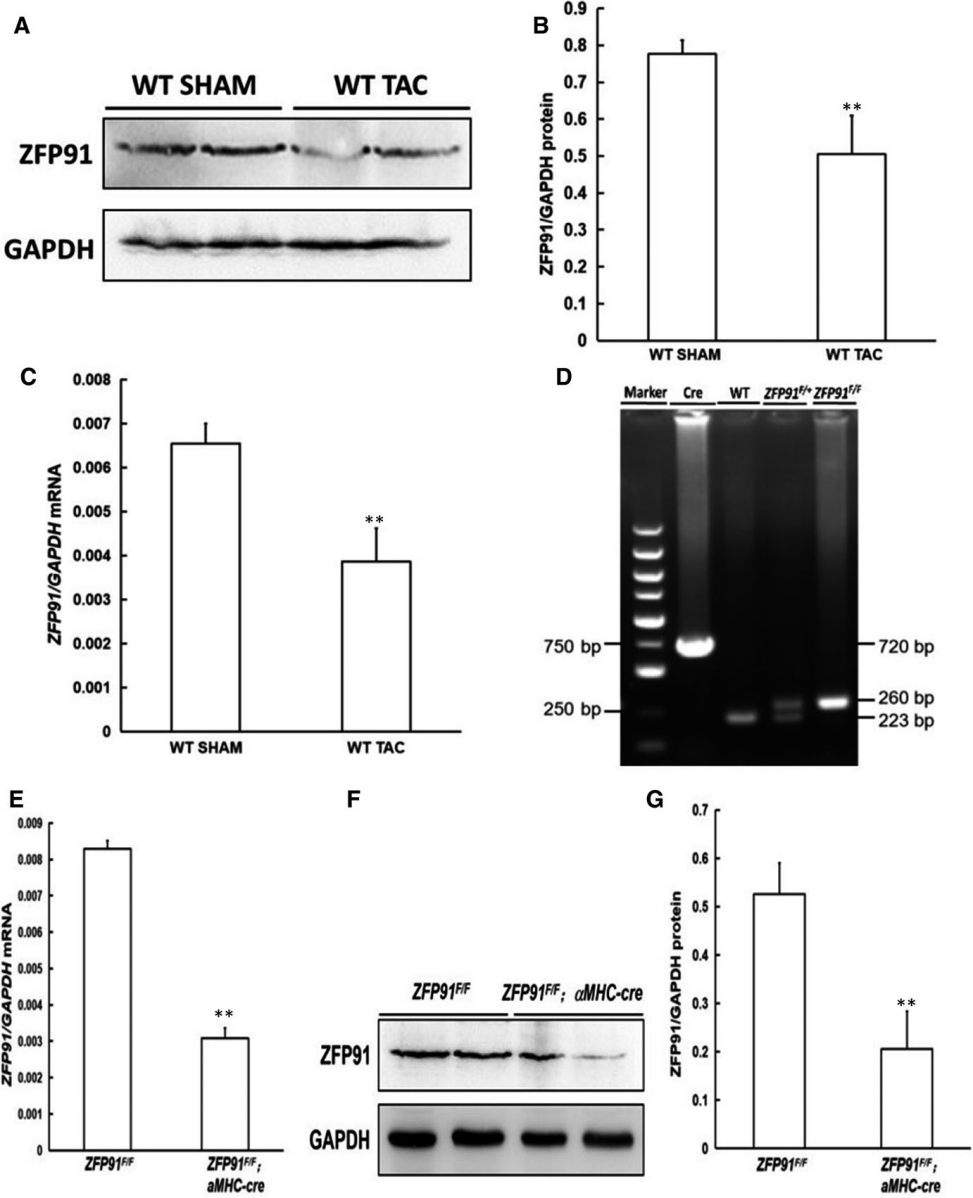
Generation of cardiomyocyte‐specific *Zfp91*‐deletion mice. A, Expression of cardiac ZFP91 protein analysed by Western blot in mice without or with TAC; B, quantitative analysis (n = 5); C, level of cardiac ZFP91 mRNA measured by real‐time PCR in mice without or with TAC (n = 5); D, genotyping of mice by PCR; E, ZFP91 mRNA level determined by real‐time PCR (n = 5); F, Western blot analysis of ZFP91 protein expression in heart; G, quantitative analysis (n = 5). WT: wild type; ZFP 91: zinc finger protein 91; SHAM: sham surgery; TAC: transverse aortic constriction; PCR: polymerase chain reaction. Data are given as means ± SEM. *P < .05 or **P < .01 vs WT or *ZFP91^F/F^* mice

Compared with the control group, we found that the expression levels of zfp91 mRNA and protein in the heart of mice with TAC after 1 month were significantly reduced (*P* < .05) (Figure 1A‐C). In order to further clarify whether zfp91 plays an important role in cardiac structure and functional homeostasis, we specifically knock out *zfp91* gene in mouse cardiomyocytes. Female *Zfp91*‐floxed mice (*Zfp91* ^F/F^) were crossed with male *aMHC‐Cre* mice to obtain *Zfp91* ^F/+^; *αMHC‐Cre* mice. Mice were genotyped by PCR (Figure 1D), and loss of Zfp91 in heart tissue was confirmed by RT‐PCR and Western blot analysis. The results revealed that *Zfp91* mRNA and protein levels were significantly reduced in *Zfp91* ^F/F^‐deletion mice as compared to controls (*P* < .01) (Figure 1E‐G).

### 3.2 Loss of Zfp91 in cardiomyocytes accelerated cardiac dysfunction and aberrant heart remodelling after TAC

**FIGURE 2:**
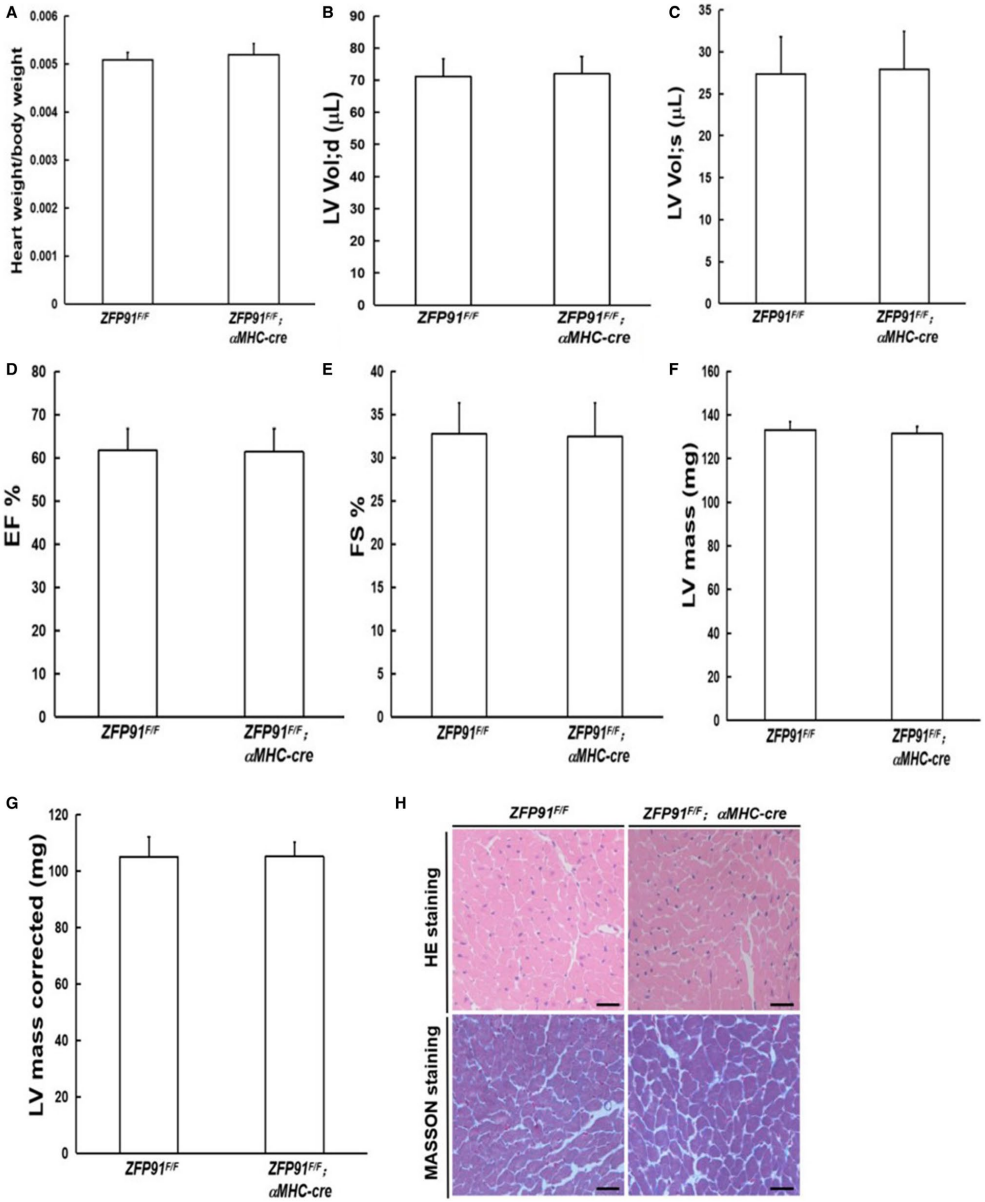
*Zfp91* loss in cardiomyocytes had no effect on heart weight to bodyweight ratio, heart function and histology in mice after 10 months. A, Heart weight to bodyweight ratio (n = 7); B‐G, echocardiography measurement (n = 7). H, Heart histology. LV: left ventricular; LV Vol;d: left ventricular end‐diastolic volume; LV Vol;s: left ventricular end‐systolic volume; EF: eject fraction; FS: fractional shortening; HE: haematoxylin and eosin. Data are given as means ± SEM

After 10 months, there were not any differences about heart weight to bodyweight ratio, as well as cardiac systolic and diastolic function between *Zfp91* ^F/F^ and *Zfp91* ^F/F^; *αMHC‐Cre* mice (*P* > .05) (Figure 2A‐G). Additionally, HE and MASSON staining results showed that the heart histology in *Zfp91* ^F/F^ mice did not change significantly as compared to *Zfp91* ^F/F^ *; αMHC‐Cre* mice (Figure 2H). Therefore, we used aortic constriction to increase pressure load of the mouse heart, and observed whether the absence of zfp91 in cardiomyocyte aggravates myocardial injury under the pathological stimulus.

**FIGURE 3:**
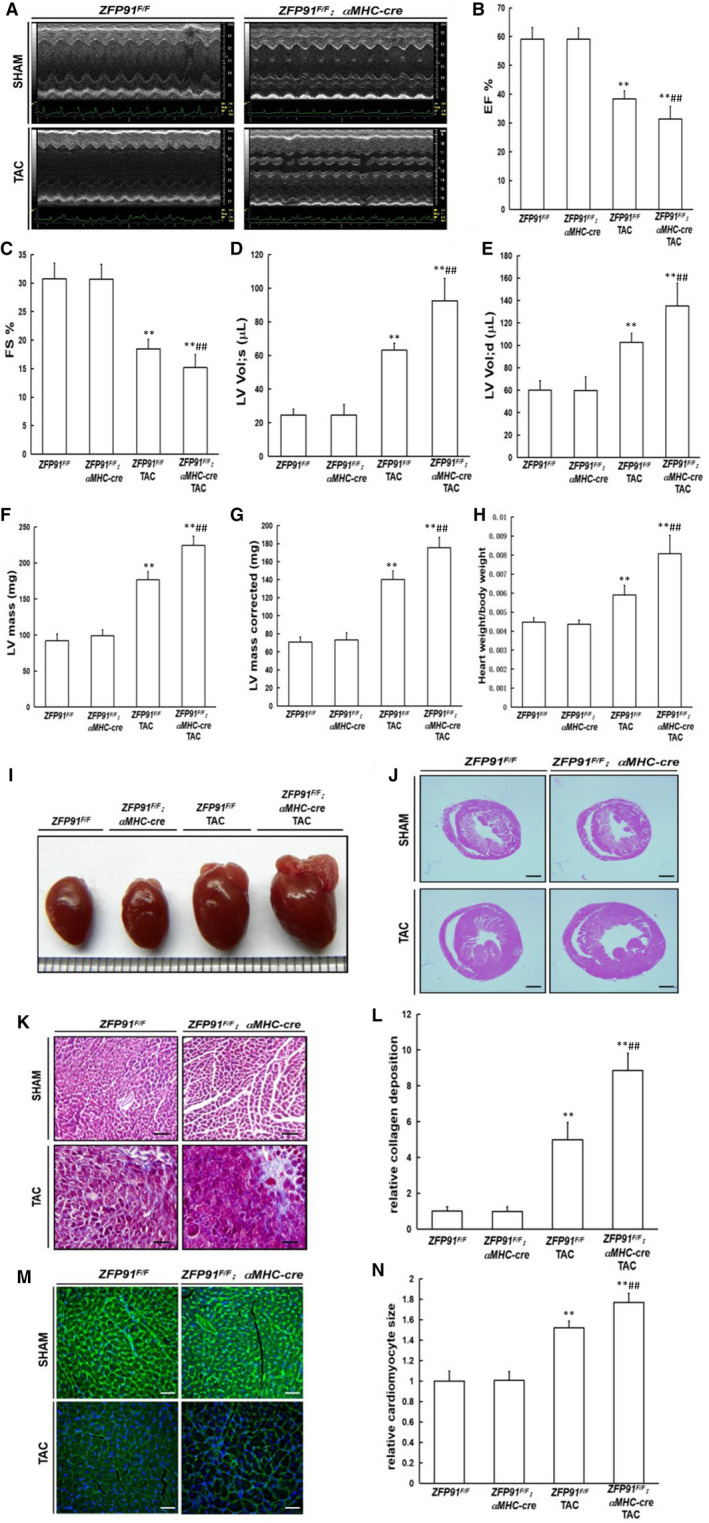
Loss of *Zfp91* in cardiomyocytes accelerated cardiac dysfunction and aberrant heart remodelling after TAC operation. A‐G, Echocardiography measurement (n = 8); H, heart weight to bodyweight ratio (n = 8); I, gross heart morphology; J, HE staining; K, Masson staining; L, quantitative analysis (n = 5); M, WGA staining; N, quantitative analysis (n = 5). WGA: wheat germ agglutinin. Data are given as means ± SEM. **P < .01 vs *ZFP91^F/F^* or *ZFP91^F/F^; αMHC‐Cre* mice; ^#^ *P *< .05 or ^##^ *P *< .01 vs *ZFP91^F/F^* mice with TAC

Six weeks after TAC surgery, echocardiography measurement indicated that heart systolic function (EF and FS) was markedly impaired in control TAC mice and in *Zfp9*1‐deletion TAC mice as compared to control mice without TAC surgery (*P* < .01) (Figure 3A‐C). Furthermore, loss of zfp91 notably decreased EF and FS values, and greatly increased the left ventricular internal diastolic and systolic volume, LV mass and LV mass (corrected) compared with control TAC mice (*P* < .01) (Figure 3A,D‐G). Additionally, the heart weight to bodyweight ratio was raised significantly in *Zfp91*‐deletion TAC mice as compared to control mice with or without TAC surgery (*P* < .01) (Figure 3H). Last, histological analysis displayed markedly increased general heart size, fibrosis area and cardiomyocyte size in *Zfp91*‐deletion TAC mice as compared to control mice with or without TAC surgery (*P* < .01) (Figure 3I‐N).

Taken together, these results indicated that genetic ablation of Zfp91 dramatically led to pathological heart remodelling and heart dysfunction after TAC surgery. Thus, Zfp91 may function to protect against myocardial damage in the heart under pressure overload condition.

### 3.3 mRNA sequencing analysis of heart tissue 1 week after TAC in mice

**FIGURE 4:**
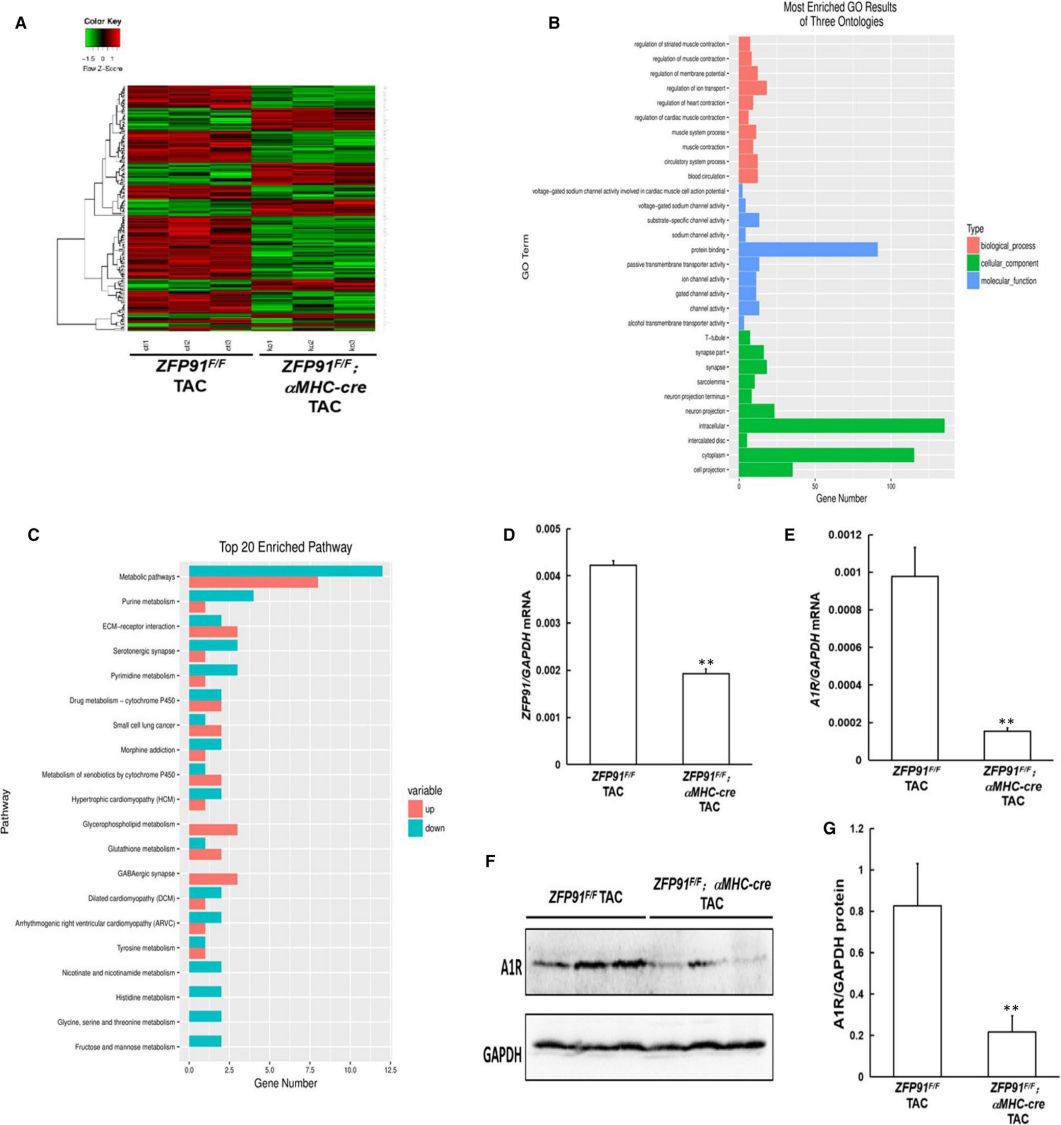
mRNA sequencing analysis of heart tissue 1 week after TAC surgery in mice. A, heat map; B, GO analysis; C, KEGG pathway analysis; D, ZFP91 mRNA level measured by real‐time PCR (n = 5); E, A1R mRNA level measured by real‐time PCR (n = 5); F, Western blot analysis of cardiac A1R protein expression. G, quantitative analysis (n = 5). A1R: adenosine A1 receptor; GO: Gene Ontology; KEGG: Kyoto Encyclopedia of Genes and Genomes. Data are given as means ± SEM. ^**^ *P *< .01 vs *ZFP91^F/F^* mice with TAC

According to heat map shown in Figure 4A, there were 201 differential mRNAs in heart tissues of *Zfp91*‐deletion TAC mice as compared to the control TAC mice (*P* < .05 or *P* < .01). Among them, 133 down‐regulated mRNAs were found in heart tissue of *Zfp91*‐deletion mice with TAC, and 68 mRNAs of heart tissues were up‐regulated (*P* < .05 or *P* < .01).

Next, we analysed Gene Ontology (GO) information from biological process (BP), cellular component (CC) and molecular function (MF) categories. In the BP aspect, muscle system process, regulations of heart contraction and cardiac muscle contraction may be the key BPs involving in the development of *Zfp91* loss–induced cardiac hypertrophy (*P* < .01). In the CC aspect, protein binding, channel activity and substrate‐specific channel activity may take an important role in the pathology of *Zfp91* loss–induced cardiac hypertrophy (*P* < .01). In the MF aspect, intracellular, cytoplasm and cell projection may play a great role in development of *Zfp91* loss–induced cardiac hypertrophy (*P* < .01) (Figure 4B). Additionally, Kyoto Encyclopedia of Genes and Genomes (KEGG) pathway was also under consideration in our study. We found mRNAs related to hypertrophic cardiomyopathy were changed significantly (*P* < .01) (Figure 4C).

From analysis of heat map, Adora1 mRNA was significantly down‐regulated in heart of *zfp91*‐deletion mice with TAC as compared to control TAC mice (*P* < .01). RT‐PCR confirmed that *zfp91*‐deletion mice with TAC displayed significant reduction of *zfp91* and *adora1* mRNAs in heart of *zfp91*‐deletion mice with TAC (*P* < .01) (Figure 4D,E). From bioinformatics analysis, Adora1 belonged to cell projection, and muscle skeletal and cardiovascular systems. Last, Western blot analysis also showed that Adora1 was notably decreased at protein level in heart of *zfp91*‐deletion mice with TAC as compared to control TAC mice (*P* < .01) (Figure 4F,G).

In summary, these results indicated that *adora1* may be the major target gene of ZFP91.

### 3.4 Selective Adora1 antagonist abolished reversion of ISO‐induced cardiomyocyte hypertrophy by Zfp91 overexpression

**FIGURE 5:**
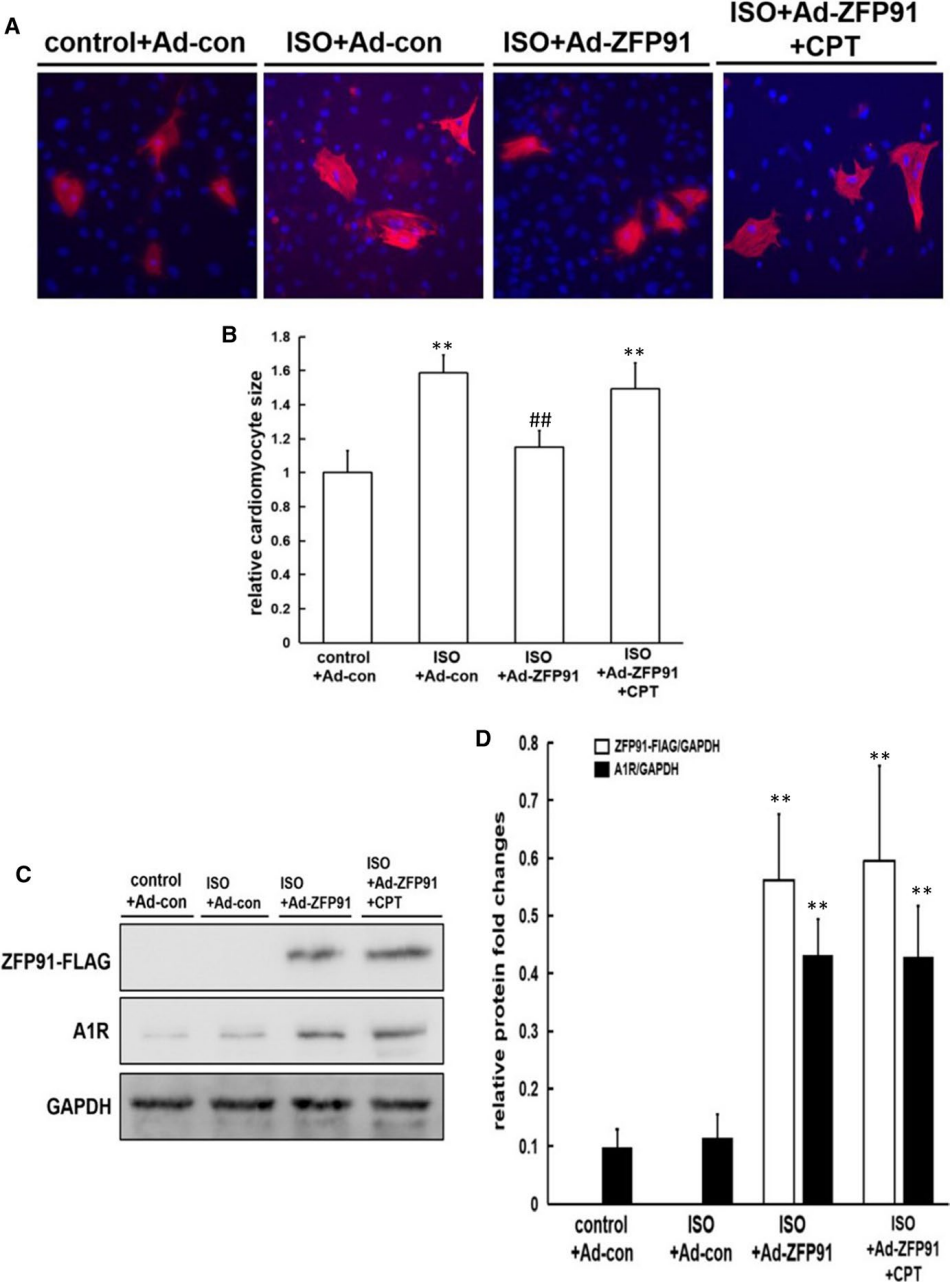
Selective Adora1 antagonist abolished reversion of ISO‐induced cardiomyocyte hypertrophy by *ZFP91* overexpression. A, cTnT staining (red) of cardiomyocytes and the cell nucleus (blue). B, Quantitative analysis (n = 5); C, Western blot analysis of ZFP91‐FLAG and A1R protein expression; D, quantitative analysis (n = 5). ISO: isoproterenol; cTnT: cardiac troponin T; Ad: adenovirus; CPT: 8‐Cyclopentyl‐1,3‐dimethylxanthine. Data are given as means ± SEM. ^**^ *P *< .01 vs control group; ^#^ *P *< .05 or ^##^ *P *< .01 vs ISO group

Next, we further investigated the role of overexpressing *Zfp 91* gene in primary neonatal rat cardiomyocytes (PNRCs) interfered with ISO. After 24 hours, the sizes of PNRCs in ISO group were significantly large as compared to those in control group (*P* < .01). However, after simultaneous transfection with Ad‐ZFP91‐Flag, the sizes of PNRCs were obviously small as compared to those of ISO group, which was abolished by Adora1 antagonist 8‐Cyclopentyl‐1,3‐dimethylxanthine (CPT) (*P* < .01) (Figure 5A,B).

Meanwhile, success overexpression of ZFP91 protein in PNRCs was confirmed by Western blot shown in Figure 5C,D. Also, expression of Adora1 protein was markedly increased after transfection with Ad‐ZFP91‐Flag in PNRCs (Figure 5C,D).

In short, overexpression of ZFP91 alleviated cardiomyocyte hypertrophy induced by ISO, which may be closely associated with Adora1 up‐regulation.

### 3.5 Interaction between transcription factor ZFP91 and adora1 promoter

**FIGURE 6:**
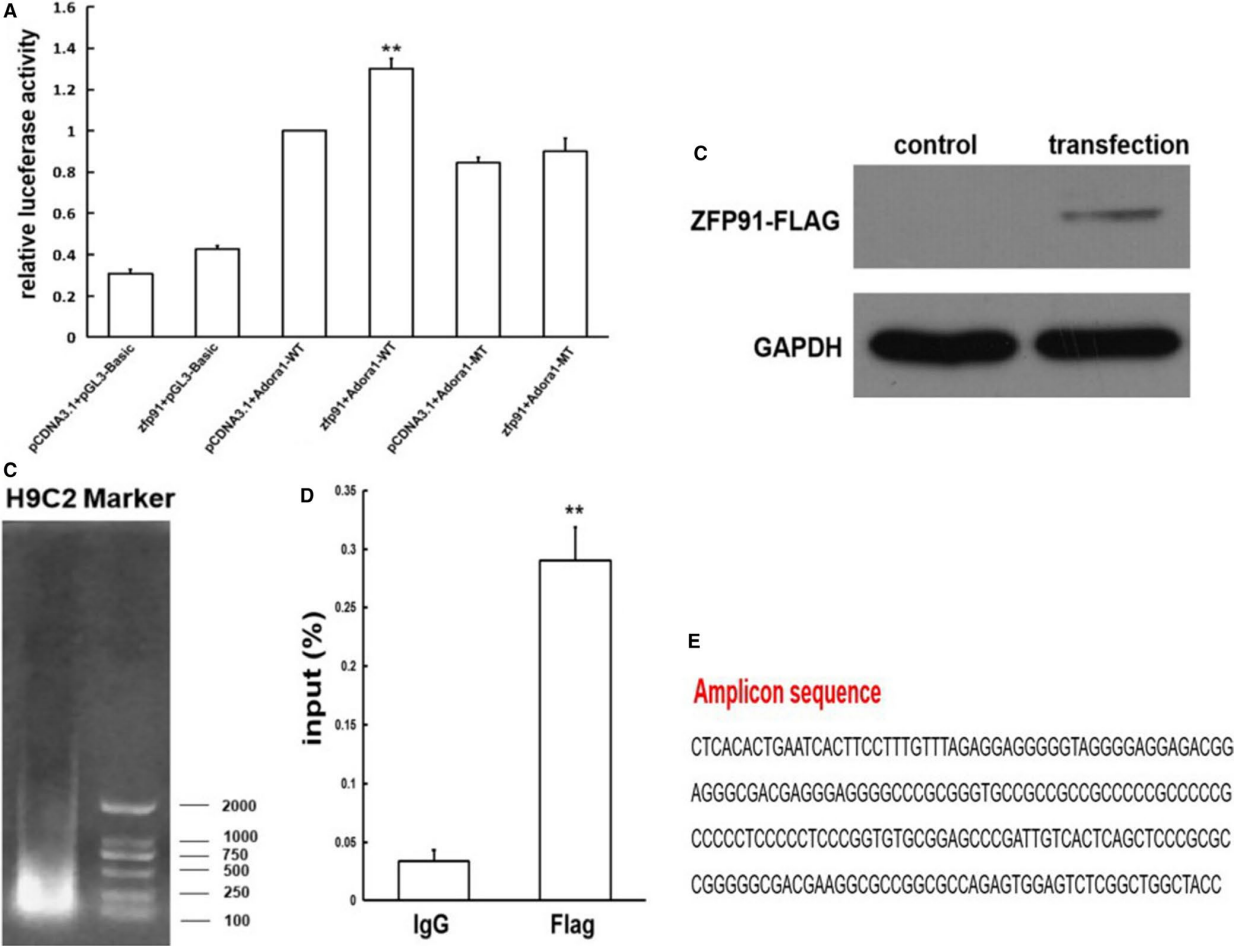
Interaction between transcription factor zfp91 and *adora1* promoter. A, Dual‐luciferase reporter assay (n = 5); B, transfection efficiency of ZFP91 gene determined by Western blot; C, PCR analysis of DNA fragments; D, qPCR analysis of the Adora1 promoter segment (n = 3); E, amplicon sequencing. Adora1: adenosine A1 receptor; WT: wild type; MT: mutant type. Data are given as means ± SEM. ^**^ *P *< .01 vs pCDNA3.1 + Adora1‐WT or IgG group

According to the information of ZFP‐related transcription factors, the possible binding site of zfp91 and *adora1* promoter was predicted on the website (http://jaspar.genereg.net/). When zfp91 and its possible binding site in *adora1* promoter were cotransfected, the dual‐luciferase activity was significantly enhanced as compared to its control group (*P* < .01). Then, the possible binding site of *zfp91* was mutated, and the result showed that the dual‐luciferase activity was not changed after cotransfection with zfp91 as compared to its control group (*P* > .05) (Figure 6A).

In order to further test interaction between zfp91 and its possible binding site in *adora1* promoter, we did ChIP‐qPCR after H9C2 cell line successfully transfected with Ad‐zfp91‐Flag, which was confirmed by Western blot (Figure 6B). Figure 6C displays that DNA products were fragmented by ultrasound for 10 minutes and their fragment size is between 100 and 500 bp. After qPCR with the primer of the Adora1 promoter segment, we found that as compared to Ig group, input value of anti‐Flag group was markedly increased, indicating that Adora1 promoter segment was enriched in zfp91‐Flag group (*P* < .01) (Figure 6D). Last, we sequenced the PCR products and found that they were part of Adora1 promoter sequences, including zfp91 binding site (Figure 6E).

Totally, these results revealed that transcription factor zfp91 can act on Adora1 promoter through its binding site.

### 3.6 Adora1 agonist reversed cardiac dysfunction and aberrant heart remodelling in *zfp91* loss mice after TAC surgery

**FIGURE 7:**
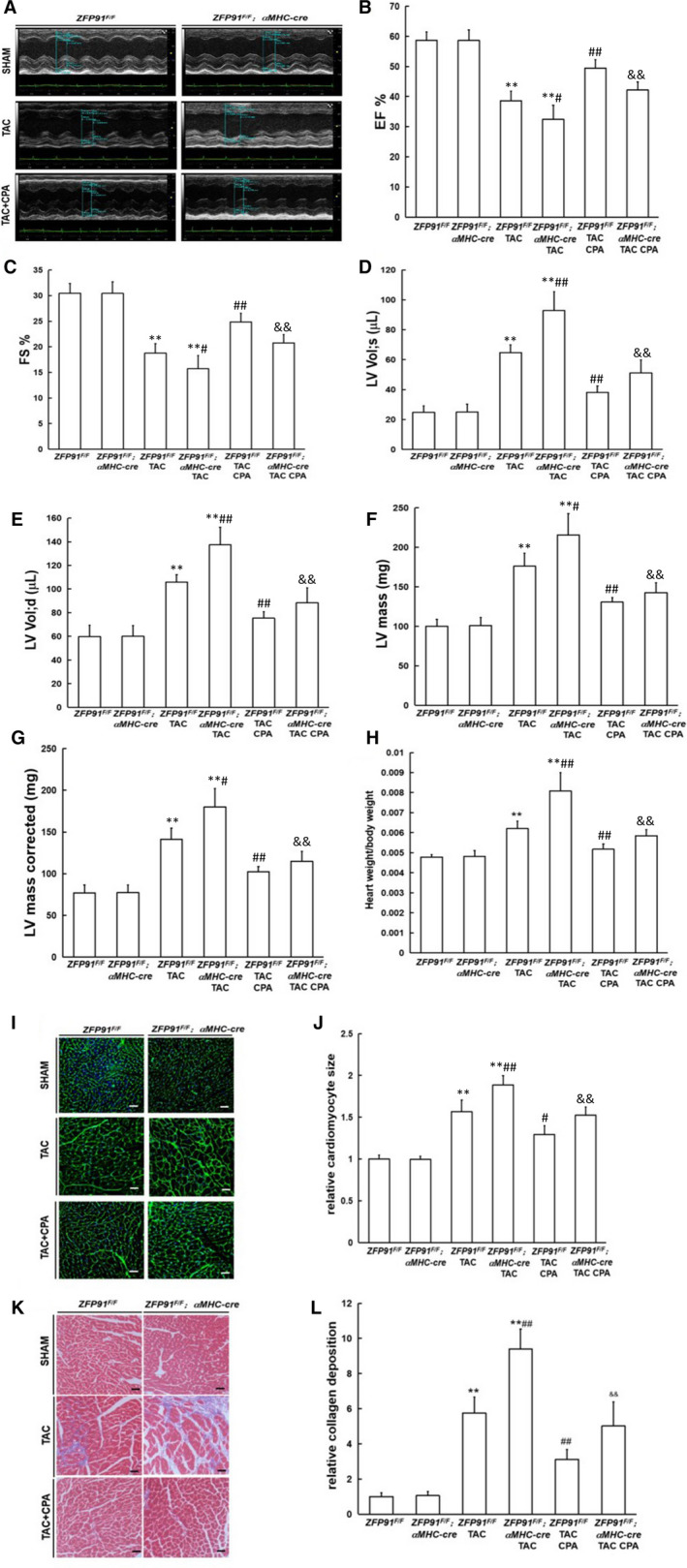
Adora1 agonist reversed cardiac dysfunction and aberrant heart remodelling in *zfp91* loss mice after TAC surgery. A‐G, Echocardiography measurement (n = 7); H, heart weight to bodyweight ratio (n = 7); I, WGA staining; J, quantitative analysis (n = 5); K, Masson staining; L, quantitative analysis (n = 5). CPA: N6‐Cyclopentyladenosine. Data are given as means ± SEM. **P < .01 vs *ZFP91^F/F^* or *ZFP91^F/F^; αMHC‐Cre* mice; ^#^ *P *< .05 or ^##^ *P *< .01 vs *ZFP91^F/F^* mice with TAC; ^&^ *P *< .05 or ^&&^ *P *< .01 vs *ZFP91^F/F^; αMHC‐Cre* mice with TAC

Next, we used potent selective Adora1 agonist N6‐Cyclopentyladenosine (CPA) to treat *zfp91* loss mice after TAC for 6 weeks. After *zfp91*‐deletion TAC mice and control TAC mice treated with CPA, heart systolic and diastolic dysfunction, LV mass and LV mass (corrected) were greatly improved (*P* < .05 or *P* < .01). Meanwhile, heart weight to bodyweight ratio, cardiomyocyte size and heart fibrotic area were significantly decreased in *zfp91*‐deletion TAC mice and control TAC mice after treated with CPA (*P* < .05 or *P* < .01) (Figure 7A‐L).

In summary, these results suggested that selective Adora1 agonist can rescue the phenotype of heart dysfunction and adverse heart remodelling in *zfp91* loss mice under pressure overload.

## 4 DISCUSSION

In the present study, we provided the first genetic evidence that ablation of *zfp91* significantly accelerated heart dysfunction and adverse pathological heart remodelling after TAC surgery. *Zfp91* overexpression reversed ISO‐induced cardiomyocyte hypertrophy, which was abolished by Adora1 antagonist CPT. Then, dual‐luciferase reporter and ChIP‐qPCR assays demonstrated that transcription factor zfp91 can directly act on *Adora1* promoter through its binding site. Last, selective Adora1 agonist can greatly improve the phenotype of adverse heart remodelling in *zfp91*‐deletion mice after TAC operation. These results suggested that zfp91‐mediated *Adora1* mRNA and protein expressions were involved in the development of cardiac hypertrophy under the pressure overload status.

Unoki and his colleagues have reported that the new *zfp91* gene for the first time was found in these patients with acute myeloid leukaemia and it expressed highly in most patients' leukaemia cells. Its gene products were located in the nucleus and showed the characteristics of transcription factors (including five zinc finger domains, leucine zipper and several nuclear localization signals). In vitro, they found that zfp91 may play an important role in cell proliferation and anti‐apoptosis. When zfp91 expression was inhibited by antisense oligonucleotide, the cell proliferation was decreased and cell apoptosis was increased.^17^ Jin and his co‐workers found that zfp91 protein was overexpressed in these patients with colon cancer. By means of ChIP, EMSA gel migration and luciferase reporter assays, it was found that ZFP91 could cooperate with NF‐κB/p65 to transcribe hypoxia inducible factor‐1 to regulate development of colon cancer.^18^ In our study, we found that transcriptional factor zfp91 can directly regulate *Adora1* mRNA and protein expressions by dual‐luciferase reporter and ChIP‐qPCR methods. Conclusionally, Zfp91 as a transcription factor may regulate the expressions of multiple genes.

Cardiac myocytes can produce adenosine, and there are adenosine receptors (including A1, A2 and A3 receptor subtypes) in the heart. Adenosine has a high affinity for A1 receptor.^19^, ^20^ Matherne and his team overexpressed Adora1 in mice, which significantly reduced infarct area and improved heart systolic and diastolic dysfunction.^21^ Kitakaze et al used phenylephrine, angiotensin II and isoproterenol to stimulate cardiomyocytes hypertrophy in neonatal rat in vitro, and found that using adenylate analogue to activate Adora1 reduced cardiomyocyte size. Then, through using adenylate analogue to treat TAC mice, it was found that it can improve cardiac hypertrophy and dysfunction.^22^ Recently, Devaux's team found that Adora1 agonist can reduce myocardial hypertrophy, interstitial fibrosis and oxidative stress in mice induced by phenylephrine stimulation.^23^ Totally, long‐term sustained activation of β‐adrenergic receptor played a key role in development of pathological ventricular remodelling and heart failure, and activation of adenylate signalling pathway exerted antagonistic effect. Thus, in our present study significant reduction of *Adora1* mRNA and protein expressions in *zfp91* loss mice was closely associated with heart dysfunction and adverse cardiac remodelling under pressure overload condition, which were reversed by selective Adora1 agonist intervention. Furthermore, it was also validated that in vitro test Adora1 antagonist CPT abolished reversion of ISO‐induced cardiomyocyte hypertrophy by ZFP91 overexpression.

In summary, we demonstrated that zfp91 may transcriptionally regulate *Adora1* mRNA expression in heart, which mainly regulated myocardial homeostasis under the status of pressure overload. It will provide a new way for treating myocardial hypertrophy.

## CONFLICT OF INTEREST

The authors declare that they have no conflict of interest.

## AUTHOR CONTRIBUTIONS

**Xiangqi Wu:** Data curation (equal); funding acquisition (equal); investigation (equal); writing‐original draft (equal). **Wei You:** Data curation (equal); investigation (equal). **Zhiming Wu:** Investigation (equal). **Fei Ye:** Conceptualization (equal); funding acquisition (equal); writing‐review and editing (equal). **Shaoliang Chen:** Conceptualization (equal); supervision (equal).

## Data Availability

The data that support the findings of this study are available from the corresponding author upon reasonable request.
